# Ayres’ Hernial Truss

**Published:** 1875-06

**Authors:** 


*Ayres' Hernial Truss.*

This mechanical device for hernial support has received the endorsement of some of the most eminent surgeons of the South. Mr. Ayres' is a Virginian, and his trusses are manufactured in Richmond. He makes a beautiful instrument, the special merits of which have been acknowledged by every one who has used one. One of our friends is now wearing one which gives the greatest comfort possible, and one which meets the wants of our friend, especially as a hernial supporter. We hope an agency will be established in Atlanta and that Ayres' truss may be tested fully by the profession.

